# Farsi version of the CLEFT-Q: translation, cultural adaptation process and reliability

**DOI:** 10.1186/s12903-021-01957-7

**Published:** 2021-11-19

**Authors:** Shabnam Ajami, Shiva Torabi, Samaneh Dehghanpour, Maryam Ajami

**Affiliations:** 1 Orthodontics Research Center, School of Dentistry, Shiraz University of Medical Sciences, Shiraz, Iran; 2 Department of Orthodontics, School of Dentistry, Shiraz University of Medical Sciences, Shiraz, Iran; 3 Student Research Committee, School of Dentistry, Shiraz University of Medical Sciences, Shiraz, Iran; 4 San Francisco, USA

**Keywords:** CLEFT-Q, Orofacial cleft, Health-related quality of life, Questionnaires and surveys, Result reliability

## Abstract

**Background:**

The purpose of this study was the translation and cultural adaptation of the CLEFT-Q to Farsi and evaluating the reliability of it.

**Methods:**

The English version of the CLEFT-Q was translated to Farsi following the guidelines set forth by the International Society for Pharmacoeconomics and Outcomes Research (ISPOR). To calculate the reliability, 50 participants filled out the Farsi version of the questionnaire twice at 2-week intervals.

**Results:**

The difficulties during the translation and cultural adaptation process were as follows: 7.56% of items from the independent forward translations, 62.18% of items from the comparison between two forward translations, and 21% of items from the comparison between post-back translation and the original version. The internal consistency and stability of the Farsi version of the CLEFT-Q were 0.979 and 0.997, which both were categorized as excellent.

**Conclusion:**

The Farsi version of the CLEFT-Q is a valid and reliable tool currently available for Farsi-speaking families around the world.

**Supplementary Information:**

The online version contains supplementary material available at 10.1186/s12903-021-01957-7.

## Background

One of the most common congenital abnormalities that affect many aspects of individuals’ lives is cleft lip and/or palate (CL/P) [1]. Various rates of incidence have been reported in different regions of Iran [2–4]. The types of orofacial clefts have been described based on their locations and extensions [5].

CL/P can negatively affect patients and their families in many ways like oral health-related quality of life (OHRQoL), social well-being, facial symmetry/expression, speech, and psychological problems [6, 7]. For many years, the clinician-reported outcome was the usual way to assess CL/P treatment [8, 9]. Patient-reported outcome (PRO) instruments provide a better understanding of the impact and effectiveness of the medical procedures from patients’ viewpoints for the clinicians and decision-makers [10]. The lack of a comprehensive questionnaire specified for patients with CL/P to evaluate the quality of their lives has been described in the literature [11, 12].

CLEFT-Q is a cleft-specific patient-reported outcome (PRO) instrument to assess the treatment results in children and young adults (8–29 years) with CL/P [13]. This questionnaire is a comprehensive cross-cultural tool with a CL/P focus consists of three main domains of appearance, health-related quality of life, and facial function divided into 13 scales as follows: the appearance of the face, nose, nostrils, teeth, lips, jaws, cleft scar; speech, psychological, social and school function; speech distress, and eating/drinking [13]. The content, construct and criterion validity, and reliability of the questionnaire have been evaluated in previous studies [13–16].

As a result, CLEFT-Q is a unique tool for the research field of CL/P. The goal of this study was the translation, cultural adaptation, and evaluation of the reliability of the CLEFT-Q for CL/P patients in Farsi-speaking families.

## Methods

All experimental protocols and methods in the present study were ethically approved by the Ethical Committee of Shiraz University of Medical Sciences (IR.SUMS.DENTAl.REC.1400.30). Each participant and their families signed the informed consent form to answer the questionnaire.

All methods were carried out according to the instructions of the CLEFT-Q team, based at McMaster University, Canada, which was the guideline of the International Society for Pharmacoeconomics and Outcomes Research (ISPOR) for the translation and cultural adaptation of instruments [17].

### Translation and cultural adaptation process

Translation and cultural adaptation of the CLEFT-Q into Farsi took place between November 2017 and November 2018. All participants of this study were patients of the cleft lip and palate clinic, Orthodontics research center, Shiraz University of Medical Sciences, who were at the clinic for their routine treatment procedures. The participants of this study were chosen conveniently.

**Fig. 1:**
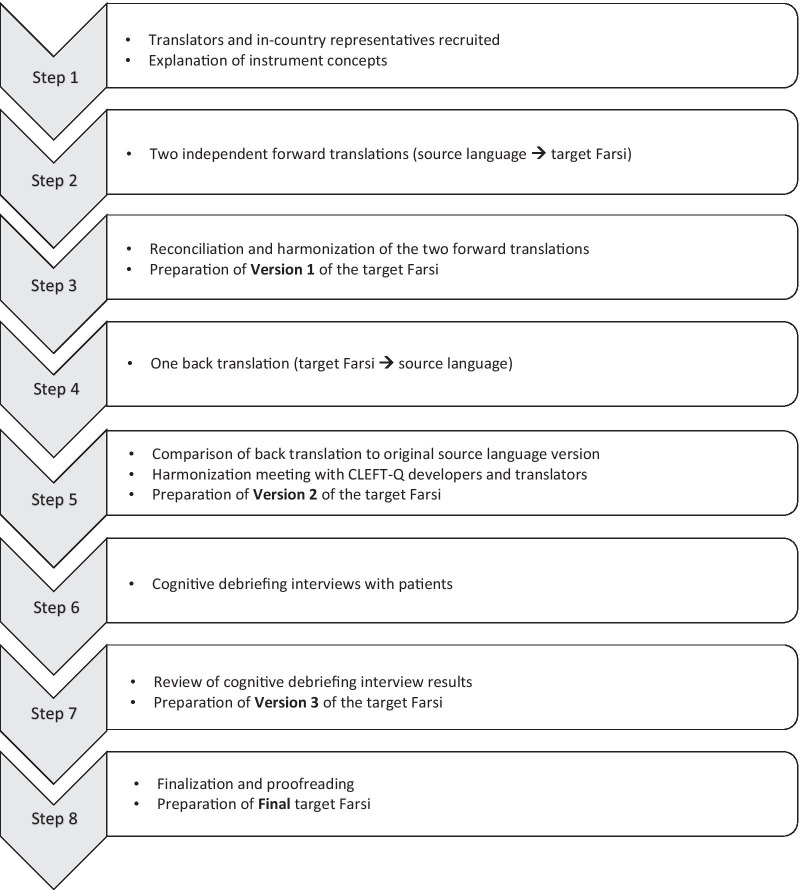
Translation and cultural adaptation steps for the CLEFT-Q

The translation and cultural adaptation process comprised of eight steps (Fig. 1):

1. Recruiting translators
  Three individuals who were fluent in English (source language) and Farsi (target language) participated in the process. Two individuals one orthodontist and one dentist who served as forward translators (English to Farsi) whose mother tongue was Farsi and fluent in English (S.D and Sh.A), and one dentist who was the back translator (Farsi to English) whose mother tongue was Farsi but has lived in an English-speaking country for many years (M.A). A member of the CLEFT-Q team was assigned as the project manager. The translators were instructed to provide feedback on any difficult items due to the construction, language, or cultural differences [17].
2. Independent forward translation
  The forward translators separately translated the questionnaire (during 2 months period). Each translator filled out a Microsoft Excel (2016) work-sheet named “items difficult to translate”.
3. Preparation of version 1 of the target language
  The two forward translations were compared together, and the discrepancies were identified. After the reconciliation and harmonization of the translated versions, version 1 of the Farsi questionnaire was prepared.
4. Post-back translation
  Version 1 of the questionnaire was sent to the back translator for the post-back translation (Farsi to English) (during 2 months period). The back translator did not see or review the original version of the CLEFT-Q.
5. Preparation of version 2 of the target language
  The forward translators compared the back translation and the original version and entered the discrepancies in a Microsoft Excel (2016) work-sheet named “post-back translation comparison” (during 2 weeks period).
  In this stage, all documents were sent to the project manager to solve the controversies. The project manager responded in terms of the semantic and idiomatic equivalence of the original version and the target language version (during 2 weeks period). After resolving the problems and final harmonization, version 2 of the questionnaire in the target language was prepared.
6. Cognitive debriefing interviews with patients
  The cognitive interviews with six patients were performed with the target patient population (mean age = 9.83 years) to determine the quality of the translation. One of the members of the research team (Sh.T) arranged the interviews at the clinic (during 4 months period). The cognitive debriefing interviews were conducted using the “think aloud” approach (patients verbalized what the question was asking them). Each participant answered the CLEFT-Q verbalizing each question and what they thought it was asking [18, 19]. Recognizing difficult items for participants was the result of this process. When any problems were found, the interviewer described the meaning to the participant, and the participant was asked to offer a substitute word/phrase to improve comprehension. The results were entered in a Microsoft Excel (2016) work-sheet named “cognitive interview reports”.
7. Preparation of version 3 of the target language
  After the cognitive debriefing of the questionnaire according to the interview results, version 3 of the target language was prepared.
8. Preparation of final target language version
  In this stage, all the reports and version 3 was sent to the project manager for finalization and proofreading. After careful final checks, the final version was prepared in Farsi.

### Reliability

In this study, the internal consistency of the questionnaire and its scales, as well as the stability, were measured. To evaluate the internal consistency, 50 patients (mean age = 12.96 years) with cleft lip and palate filled out the questionnaire. The test–retest method was used to calculate the stability of the CLEFT-Q in which the 50 participants answered the questionnaire for the second time at 2-week intervals. The results were interpreted as follow: “_ > 0.9—Excellent, _ > 0.8—Good, _ > 0.7—Acceptable, _ > 0.6—Questionable, _ > 0.5—Poor, and_ < 0.5—Unacceptable” [20]. Table 1 demonstrates the characteristics of the participants of this study.

**Table 1:** Characteristics of patients participating in this study (N = Number)

	Reliability assessment (N = 50)	Cognitive debriefing interviews (N = 6)
*Age*	N (%)	N (%)
8–11	23 (46.0)	6 (100.0)
12–15	12 (24.0)	0 (0.0)
16–19	11 (22.0)	0 (0.0)
20–23	2 (4.0)	0 (0.0)
24–29	2 (4.0)	0 (0.0)
*Gender*		
Male	31 (62.0)	4 (66.6)
Female	19 (38.0)	2 (33.4)
*Cleft type*		
Cleft lip/cleft lip and alveolus	0 (0.0)	0 (0.0)
Cleft lip and palate	50 (100.0)	6 (100)
Cleft palate only	0 (0.0)	0 (0.0)

### Statistical analysis

Cronbach’s alpha coefficient was used to calculate the internal consistency. The test–retest reliability was determined using the intraclass correlation coefficient (ICC). The results from the analyses were considered to be significant at *P* < 0.001. PASW SPSS software for Windows version 18.0 (SPSS Inc., Chicago, IL, USA) was used for data analysis.

## Results

### Translation process

After the independent forward translation, the translators described that 9 items out of a total of 119 items (7.56%) were difficult to translate. For instance, items such as “How much do you like the color of your cleft lip scar?” and “I feel upset when I am not understood” were difficult to translate, as the use of the verb “like” with the word “scar”, and “I am not understood” are phrases not commonly used in Farsi. The comparison of the two forward translations revealed the inconsistency of 74 items (62.18%) which were related to the wording or phrasing of the items.

The comparison between the original version and the back-translation version showed that in 25 items of the questionnaire (21%), semantic, idiomatic, or conceptual equivalence was not achieved. The Additional file 1: Table S1 shows some of these items in more detail (see Additional file 1: Table S1). None of the participants in cognitive debriefing interviews reported any difficulties.

### Reliability

Table 2 shows the results of the internal consistency of the Farsi version of the CLEFT-Q which was 0.979 overall. The results were categorized as excellent on each scale except for the “drinking and eating” scale. The outcomes of test–retest reliability are indicated in Table 3. The overall stability was 0.997. The categorization of the stability results was similar to the results of internal consistency in all scales.

**Table 2:** Results of the internal consistency of the Farsi version of the CLEFT-Q

Scale of the CLEFT-Q	Cronbach’s alpha	Interpretation	Number of items
Appearance of the face	0.958	Excellent	9
Appearance of the nose	0.979	Excellent	12
Appearance of the nostrils	0.981	Excellent	6
Appearance of the teeth	0.967	Excellent	8
Appearance of the lips	0.966	Excellent	9
Appearance of the lip scar	0.983	Excellent	7
Appearance of the jaws	0.962	Excellent	7
Speech function	0.955	Excellent	12
Speech distress	0.932	Excellent	10
Psychological function	0.981	Excellent	10
School function	0.927	Excellent	10
Social function	0.918	Excellent	10
Drinking and eating	0.798	Acceptable	9
Total	0.979	Excellent	119

**Table 3:** Results of the test–retest stability of the Farsi version of the CLEFT-Q

Scale of the CLEFT-Q	ICC	*P* value	Interpretation	Number of items
Appearance of the face	0.989	S	Excellent	9
Appearance of the nose	0.993	S	Excellent	12
Appearance of the nostrils	0.943	S	Excellent	6
Appearance of the teeth	0.990	S	Excellent	8
Appearance of the lips	0.990	S	Excellent	9
Appearance of the lip scar	0.954	S	Excellent	7
Appearance of the jaws	0.995	S	Excellent	7
Speech function	0.996	S	Excellent	12
Speech distress	0.991	S	Excellent	10
Psychological function	0.995	S	Excellent	10
School function	0.990	S	Excellent	10
Social function	0.992	S	Excellent	10
Drinking and eating	0.757	S	Acceptable	9
Total	0.997	S	Excellent	119

S, significant (*P* < 0.001), ICC, intraclass correlation coefficient

## Discussion

The translation and cultural adaptation of the CLEFT-Q was done according to best-practice guidelines [17] and in a standard method. All scales of the questionnaire were found to be comprehensible and suitable by the participants. The Farsi version of the CLEFT-Q also showed an excellent rate of reliability.

The CLEFT-Q was cross-culturally developed for the international application of patients with different age ranges who were born with CL/P [13, 15]. The basis of the scales in the CLEFT-Q was a conceptual framework designed by in-depth interviews with 136 patients with clefts of any age across six countries [21]. Furthermore, collected data from 2434 participants, aged 8 to 29 years from 12 countries, was used to establish norm values of the CLEFT-Q [15]. Therefore, CLEFT-Q is one of the most efficient available PRO instruments specified for patients born with CL/P. As a result, developing a Farsi version of the CLEFT-Q seemed to be essential.

The ultimate aim of the translation process was to achieve a precise conceptual translation rather than a literal one, which called for a scientific methodology to achieve complete equality of the questions, instructions, and response options. The ISPOR provided a standard method to accomplish the goals of the translation and cultural adaptation process [17]. Cognitive debriefing interviews were one of the most vital steps of the translation process. The participation of a few patients certified the ease of understanding and application of the Farsi version of the CLEFT-Q [22].

No significant challenge emerged during the translation and cultural adaptation process. Constant consultation and coordination between the translators and the project manager allowed the team to follow the process accurately and scientifically. The team members anticipated that the cognitive interviews would lead to face some problems with children, especially the ones between ages 8 to 10, due to their lesser ability to read texts. Surprisingly, even the slow-readers could comprehend the items of the questionnaire easily. Several studies reported that children as young as 8 years age are capable of providing self-reported outcomes [23–25]. However, inadequate formal education of some of the children from the deprived regions and the length of the interview arose some challenges during the process.

The field-test version of the CLEFT-Q consisted of 154 items with 13 scales was translated to Colombian, Chilean, Spanish, Arabic, Dutch, Hindi, Swedish, and Turkish [22, 26]. After forward translation to Farsi, 7.54% of the items, were found difficult to translate. Only Arabic and Swedish forward translators reported a higher rate of difficulties comparing to Farsi, and the rest of the translators faced fewer problems [22, 26]. The inconsistency between the two forward translations was 62.18%. The rate of inconsistency in Turkish and Colombian translations was above the Farsi translation, while other languages translators stated lower rates [22, 26]. This relatively high rate of inconsistency was the result of slightly different approaches of the forward translators, which was raised at the consensus meeting. One of the translators was trying to keep the translation literally as possible, while the other one was to achieve the conceptual translation. Comparing the original version and back-translated version of the CLEFT-Q resulted in finding that 21% of items didn’t have semantic, idiomatic, or conceptual equivalence. This rate was more than all other languages except for Turkish [22, 26]. These various rates are the result of the significant grammatical differences between the languages and their diverse origins. None of the Farsi-speaking participants in cognitive debriefing interviews stated any problem, while some difficulties during the interviews in other languages were reported [22, 26]. This may be relevant to the fact that the Farsi version of the CLEFT-Q was translated from the final English version, while the other translations were done from the field-test version of the CLEFT-Q.

The evaluation of the reliability of the Farsi version of the CLEFT-Q demonstrated excellent internal consistency and stability. It might seem that the relatively high results of reliability were because of the multiple numbers of the questionnaire items [27]. But the outcome didn’t decrease significantly in each scale with fewer questions. The CLEFT-Q scales can operate independently (meaning only relevant scales to each specific individual can be used) [13, 28]. Therefore, the reliability of each scale, as well as the whole questionnaire, was assessed. The internal consistency and the stability of all scales were categorized as excellent except for the “drinking and eating” scale. It might be because the participants suffered from varying degrees of food regurgitation as one of the complications of palatal repair surgery.

Following one of the best available guidelines for the translation and cultural adaptation, adequate size of the sample to assess the reliability, and recruiting participants from various socio-economics status and regional cultures were the strengths of this study. A potential limitation is that the back-translator mother tongue was not English, which differed from the ISPOR recommendations. Nonetheless, it seems improbable that this slight deviation from the ISPOR guideline has influenced the quality of the translation. The fact that participants of cognitive debriefing interviews were not from a wide age range and all different kinds of CL/P is another possible limitation. However, children were more likely to report difficulties comprehending the items of the questionnaire than the older individuals. Besides, patients with cleft lip and palate usually struggle with problems that other categories of individuals with CL/P would face them too. The mentioned reasons would ensure that the participants were a representative sample.

## Conclusion

The Farsi version of the CLEFT-Q is a valid and reliable tool currently available for Farsi-speaking families around the world.

## Supplementary Information

**Additional file 1**. **Table S1**. Examples of Discrepancies between the Original English Version and Back-translated English Version.

## Data Availability

The data and materials that support the findings of this study are available from McMaster University, Canada, but restrictions apply to the availability of these data and materials, which were used under license for the current study, and so are not publicly available. Data are however available from the authors upon reasonable request and with permission of McMaster University, Canada.

## Abbreviations

CL/P: Cleft lip and/or palate
PRO: Patient-reported outcome
ISPOR: International Society for Pharmacoeconomics and Outcomes Research
ICC: Intraclass correlation coefficient
