# Supplementary material for: Farsi version of the CLEFT-Q: translation, cultural adaptation process and reliability
Source: BMC Oral Health. 2021 Nov 19;21:593. doi: 10.1186/s12903-021-01957-7 (PMC8603523; doi:10.1186/s12903-021-01957-7)
Supplement: Supplementary file 1 — Additional file 1. Table S1. Examples of Discrepancies between the Original English Version and Back-translated English Version. [file 12903_2021_1957_MOESM1_ESM.docx]

| **Additional file 1: Table S1. Examples of Discrepancies between the Original English Version and Back-translated English Version** | | | | | | | |
| --- | --- | --- | --- | --- | --- | --- | --- |
| **Scale** | **Original English Version** | **Back-translated English Version** | **Wording discrepancy (Yes/No)** | **Changing Meaning (Yes/No)** | **Comment** | **Translation Change** | **Change** |
| Appearance of Nostrils | How much do you like the size of your nostrils? | How satisfied are you with the size of your nostrils? | Yes | Yes | Not a satisfaction scale. The stem should be “like” | Yes | Used equivalent Farsi verb for “like” |
| Speech Difficulty | I get teased about my speech. | I get angry about the way I speak. | Yes | Yes | Use “teased” or “made fun of” not “angry” | Yes | Used equivalent Farsi phrase for “tease’ |
| School Function | It’s easy for me to make friends. | Finding friends is easy for me. | Yes | Yes | The phrase should be “make friends” not “find friends” | Yes | Used equivalent Farsi phrase for “making friends” |
| Psychological Function | I feel great about myself. | I feel good about myself. | Yes | Yes | Need to change the word “good” to “great” | Yes | Used equivalent Farsi word for “great” |
